# Effectiveness of pulse methylprednisolone in patients with non-human immunodeficiency virus pneumocystis pneumonia: a multicentre, retrospective registry-based cohort study

**DOI:** 10.1186/s12879-024-10151-3

**Published:** 2024-11-02

**Authors:** Yasuhiro Morimoto, Hiroki Matsui, Haruka Fujioka, Yuya Homma, Tatsuya Nagai, Ayumu Otsuki, Hiroyuki Ito, Shin-ichiro Ohmura, Toshiaki Miyamoto, Daisuke Shichi, Tomohisa Watari, Yoshihito Otsuka, Kei Nakashima

**Affiliations:** 1 Department of Pulmonology, Kameda Medical Center, 929 Higashi-cho, Kamogawa, 296-8602 Chiba Japan; 2 Division of Respiratory Diseases, Department of Internal Medicine, The Jikei University School of Medicine, Tokyo, Japan; 3 Department of Clinical Epidemiology and Health Economics, School of Public Health, the University of Tokyo, Tokyo, Japan; 4 Clinical Research Support Office, Kameda Medical Center, Kamogawa, Chiba Japan; 5 Department of Rheumatology, Seirei Hamamatsu General Hospital, Hamamatsu, Shizuoka Japan; 6 Department of Infectious Diseases and Rheumatology, Seirei Mikatahara General Hospital, Hamamatsu, Shizuoka Japan; 7 Department of Laboratory Medicine, Kameda Medical Center, Kamogawa, Chiba Japan

**Keywords:** Non-human immunodeficiency virus-infected patient, Pneumocystis pneumonia, Adjunctive corticosteroid treatment, Pulse methylprednisolone

## Abstract

**Background:**

A recent database study and meta-analysis reported that adjunctive glucocorticoid therapy reduces mortality in patients with non-human immunodeficiency virus-associated (non-HIV) *Pneumocystis jirovecii* pneumonia (PCP), having hypoxemia. However, the optimal glucocorticoid dose remains unclear. Our study aimed to evaluate the effectiveness of pulse methylprednisolone compared with mild-to-moderate steroid doses in patients with non-HIV PCP.

**Methods:**

This multicentre retrospective cohort study included adults with non-HIV PCP receiving adjunctive steroids at three Japanese tertiary care hospitals from June 2006 to March 2021. Patients were categorised into pulse methylprednisolone and mild-to-moderate dose groups. Pulse methylprednisolone involved an initial intravenous infusion of 500–1000 mg methylprednisolone daily, while the mild-to-moderate dose was lower. Primary and secondary outcomes were 30-day and 180-day mortality from treatment initiation. Patient characteristics were adjusted using propensity score analysis with overlap weighting. Subgroup analysis focused on patients with respiratory failure.

**Results:**

The study included 139 patients with non-HIV PCP: 55 in the pulse methylprednisolone group and 84 in the mild-to-moderate dose group. After adjusting for patient background, 30-day mortality (14.2% vs. 15.5%, *P* = 0.850) and 180-day mortality (33.5% vs. 27.3%, *P* = 0.516) did not differ significantly between groups. Subgroup analysis revealed no significant associations among patients with respiratory failure.

**Conclusions:**

After adjusting for patient characteristics, no difference in prognosis was observed between pulse methylprednisolone and mild-to-moderate dose groups in patients with non-HIV PCP. A mild-to-moderate dose of adjunctive corticosteroid may suffice for treating non-HIV PCP.

**Supplementary Information:**

The online version contains supplementary material available at 10.1186/s12879-024-10151-3.

## Background

*Pneumocystis jirovecii* pneumonia (PCP) is a life-threatening opportunistic infection occurring in patients with human immunodeficiency virus (HIV) infection and other immunocompromised states [1]. Owing to differences in pathophysiology, non-HIV-associated (non-HIV) PCP has been reported to cause a more severe inflammatory response and profound hypoxemia than in those with HIV [1, 2]. The mortality rates in patients with non-HIV PCP (30–60%) have been reported to be higher than those in patients with HIV (10–20%) [1]. Recent epidemiological studies have shown an increasing trend in the incidence and mortality of non-HIV PCP [2–4].

The first-line antibiotic for treating PCP is trimethoprim-sulfamethoxazole [1, 5–7]. Adjunctive steroids are recommended for patients experiencing hypoxemia, which is characterised by a partial pressure of oxygen < 70 mmHg and an alveolar-arterial oxygen gradient of ≥ 35 mmHg [5, 7]. Previous randomised controlled trials and a meta-analysis have demonstrated that adjunctive corticosteroid treatment reduces the need for ventilation and mortality in patients with HIV-PCP with such hypoxemia [8–11]. However, limited data exist on the effectiveness of adjunctive glucocorticoid therapy in patients with non-HIV PCP [12].

A nationwide administrative claims database study and a meta-analysis have reported that adjunctive glucocorticoid therapy reduces mortality in patients with non-HIV PCP having respiratory failure [13, 14]. Evidence supporting the efficacy of adjunctive glucocorticoid therapy in cases of respiratory failure is accumulating. However, the appropriate dosage of glucocorticoids has not been well studied. The nationwide database study suggested that low-dose corticosteroids (prednisone equivalents 17–36 mg/day) are associated with a lower risk of all-cause mortality in a subgroup of patients with respiratory failure [13]. In a subgroup analysis conducted in the recent meta-analysis [14], low-dose corticosteroids (equivalent to 1 mg/kg/day prednisone) were significantly associated with reduced mortality in patients with respiratory failure, while no significant association was found with high-dose corticosteroids (prednisone equivalents > 240 mg). However, these studies have limitations. The administrative claims data study lacks detailed patient severity information, such as lactate dehydrogenase and albumin levels, which have been reported to affect the prognosis of non-HIV PCP [15]. The meta-analysis only included one small observational study of 39 cases evaluating high-dose steroids without adjustment for patient background. Therefore, further large-scale research on dosage appropriateness based on real clinical practice data, with adjustments for patient background and severity information, is necessary.

In this multicentre cohort study, our objective was to evaluate the efficacy of pulse methylprednisolone compared to mild-to-moderate steroid doses in patients with non-HIV PCP. We aimed to adjust for patient background and assess the primary endpoint of 30-day mortality.

## Materials and methods

### Study setting and population

We conducted a multicentre, retrospective, observational cohort study comprising adult patients with clinically diagnosed non-HIV PCP treated with adjunctive corticosteroid therapy. The index date was defined as the initiation date of antifungal treatment for PCP. Patients without HIV, diagnosed with PCP at Kameda Medical Center, Seirei Hamamatsu General Hospital, and Sierei Mikatahara General Hospital between January 2006 and March 2021, were retrospectively enrolled (registry named RE-VISION-PCP: Registry to Provide New Evidence and Insights for the Management of Pneumocystis Pneumonia in Non-HIV-infected Patients). The study protocol was approved by the research ethics committees of Kameda Medical Center(#21-069-230801), Seirei Hamamatsu General Hospital (#3584), and Seirei Mikatahara General Hospital (#21–58). As this was a retrospective study and patient data were anonymised, the requirement for written informed consent was waived.

Based on the diagnostic guideline for non-HIV PCP and previous studies [16–19], we defined the diagnostic criteria for PCP as follows: (1) Host factors: This includes possible immunocompromised conditions other than HIV. (2) Clinical criteria: The presence of clinical symptoms consistent with PCP, such as dyspnoea, cough, fever, and hypoxemia, as well as identification of abnormal shadows (such as bilateral ground-glass opacities) on chest X-ray or chest computed tomography scans that indicate PCP. (3) Microbiological criteria: Detection of pneumocystis in respiratory specimens, such as sputum or bronchoalveolar lavage fluid, using conventional staining methods (Grocott methenamine silver or Diff-Quick staining) or deoxyribonucleic acid testing (loop-mediated isothermal amplification or polymerase chain reaction). Alternatively, elevated levels of β-D-glucan, along with a favourable response to standard therapy for PCP, can also be considered as part of the microbiological criteria. Serum concentrations of β-D-glucan were assessed using either the β-D-glucan test kit (Wako Pure Chemical Industries, Osaka, Japan) or the FUNGITEC G test MKII (Nissui Pharmaceutical, Tokyo, Japan). Elevated β-D-glucan levels were defined as > 5 pg/mL (β-D-glucan test; Wako assay) or > 20 pg/mL (FUNGITEC G-test KM assay) [20, 21]. Notably, patients with PCP who received adjunctive corticosteroid therapy or any other treatment were included in the study.

The exclusion criteria were as follows: (1) patients who did not receive any therapy and (2) patients who did not receive adjunctive corticosteroid therapy.

### Exposure defined by steroid dosage

Patients who participated in the study were grouped into two categories based on their steroid dosage: the pulse methylprednisolone group and the mild-to-moderate dose group. Pulse methylprednisolone was defined as an initial intravenous infusion of 500–1000 mg of methylprednisolone administered daily. Alternatively, the mild-to-moderate dose group was characterised by a steroid dose lower than that used in pulse methylprednisolone therapy. In the adjunctive steroid treatment of PCP for the mild-to-moderate dose group, the administration methods and durations typically follow international guidelines derived from research conducted on HIV-associated PCP [5, 7]. The treatment regimen begins with administration of prednisone, or an equivalent dose of another corticosteroid, at 40 mg twice daily for the first 5 days, followed by 40 mg once daily from Days 6 to 11, and finally, a dosage of 20 mg daily through Day 21. Notably, treating physicians have the flexibility to adjust this regimen as needed. In the pulse methylprednisolone therapy group, an initial dose of 500–1000 mg is administered for the first 3 days, after which the established protocol for HIV-associated PCP continues from Day 4 onwards [5, 7]. The entire course of steroid treatment spans 21 days, with modifications as determined by the treating physician.

### Outcomes

The primary endpoint was the 30-day mortality from the start of the treatment. The secondary endpoint was the 180-day mortality after treatment initiation. Although there are no established international guidelines that recommend specific primary endpoints for non-HIV PCP, 30-day mortality has been widely used as an outcome measure in prior studies of PCP [19, 22–24]. Specifically, while previous randomized controlled trials evaluating PCP treatments have employed 21-day and 35-day mortality as primary endpoints [22], recent studies used 30-day mortality as either a primary or secondary outcome [19, 23, 24]. Given these precedents, we selected 30-day mortality as the primary endpoint for this study.

### Data collection

The following data were retrospectively collected for each patient from their medical records: age, sex, weight, hospital, underlying diseases, immunosuppressive agents administration at diagnosis, laboratory results (platelet count; haemoglobin, serum albumin, lactate dehydrogenase, serum sodium, serum potassium, and creatinine levels; creatinine clearance), consciousness status, presence of hypotension, respiratory status (no oxygen support, oxygen support, and mechanical ventilation), initial treatment drugs (Trimethoprim-Sulfamethoxazole, pentamidine, and atovaquone), use of adjunctive glucocorticoid therapy (including pulse methylprednisolone administration), and 30- and 180-day mortality rates.

### Statistical analyses

Descriptive statistics for baseline characteristics and outcomes were obtained for each group. As this was a retrospective cohort study, prior sample size calculations were not performed, and all available populations were enrolled. There were no missing values for the variables in the patients included in the analysis. No loss to follow-up occurred for the 30-day mortality, which was the primary endpoint of the study. T-tests were used for continuous variables and χ-square tests for categorical variables to compare between groups. Propensity scores for pulse methylprednisolone were calculated to reduce the confounding effect of the application, and overlap weighting was performed using the propensity scores [25]. For propensity score calculation, a logistic regression model was constructed with pulse methylprednisolone as the dependent variable and age, sex, hospital, serum albumin levels, lactate dehydrogenase levels, respiratory status, creatinine clearance, and the presence of malignancy, interstitial pneumonia, and connective tissue disease as explanatory variables. These explanatory variables were selected by considering results of clustering by hospital, prognostic factors for non-HIV PCP [6, 15], interstitial pneumonia, and connective tissue disease complications. We assumed that pulse methylprednisolone therapy would be more likely to be selected in cases of interstitial pneumonia due to the suspicion of acute exacerbation of interstitial pneumonia as a potential differential diagnosis. This assumption is based on the common practice in Japan, where acute exacerbations of interstitial pneumonia are often treated with pulse methylprednisolone therapy. The decision to use this treatment approach is guided by the Japanese guidelines for idiopathic pulmonary fibrosis [26]. Additionally, in patients with connective tissue disease who develop PCP, there may be concurrent worsening of the underlying connective tissue disease, including exacerbation or the onset of connective tissue disease-related interstitial pneumonia. These patients may potentially benefit from pulse methylprednisolone in terms of prognosis [27, 28]. Therefore, the presence of connective tissue disease was included as an explanatory variable.

The balance of variables between the groups was evaluated using the standardised mean difference (SMD) between the variables. As per previous studies [29], the groups were considered balanced when the SMD was less than 0.1. Statistical analyses were conducted using the R software (version 4.3.0; R Development Core Team).

### Subgroup analysis

The same methodology described previously was also used for the subgroup analysis, which was limited to patients with respiratory failure. Respiratory failure was defined as respiratory status requiring oxygen support or mechanical ventilation. This analysis was conducted based on prior studies demonstrating the benefits of adjunctive corticosteroid therapy in patients with non-HIV PCP and hypoxemia [13, 14].

### Sensitivity analysis

To confirm the robustness of the analysis, we conducted a sensitivity analysis using the log-rank test and Cox proportional hazards analysis to determine the hazard ratio (HR) of pulse methylprednisolone for mortality, with the control being mild-to-moderate dose therapy. For the adjustment of patient background in the Cox proportional analysis, we applied overlap weighting based on propensity scores, accounting for age, sex, hospital, serum albumin levels, lactate dehydrogenase levels, respiratory status, creatinine clearance, and the presence of malignancy, interstitial pneumonia, and connective tissue diseases, as done in the primary analysis.

## Results

**Fig. 1:**
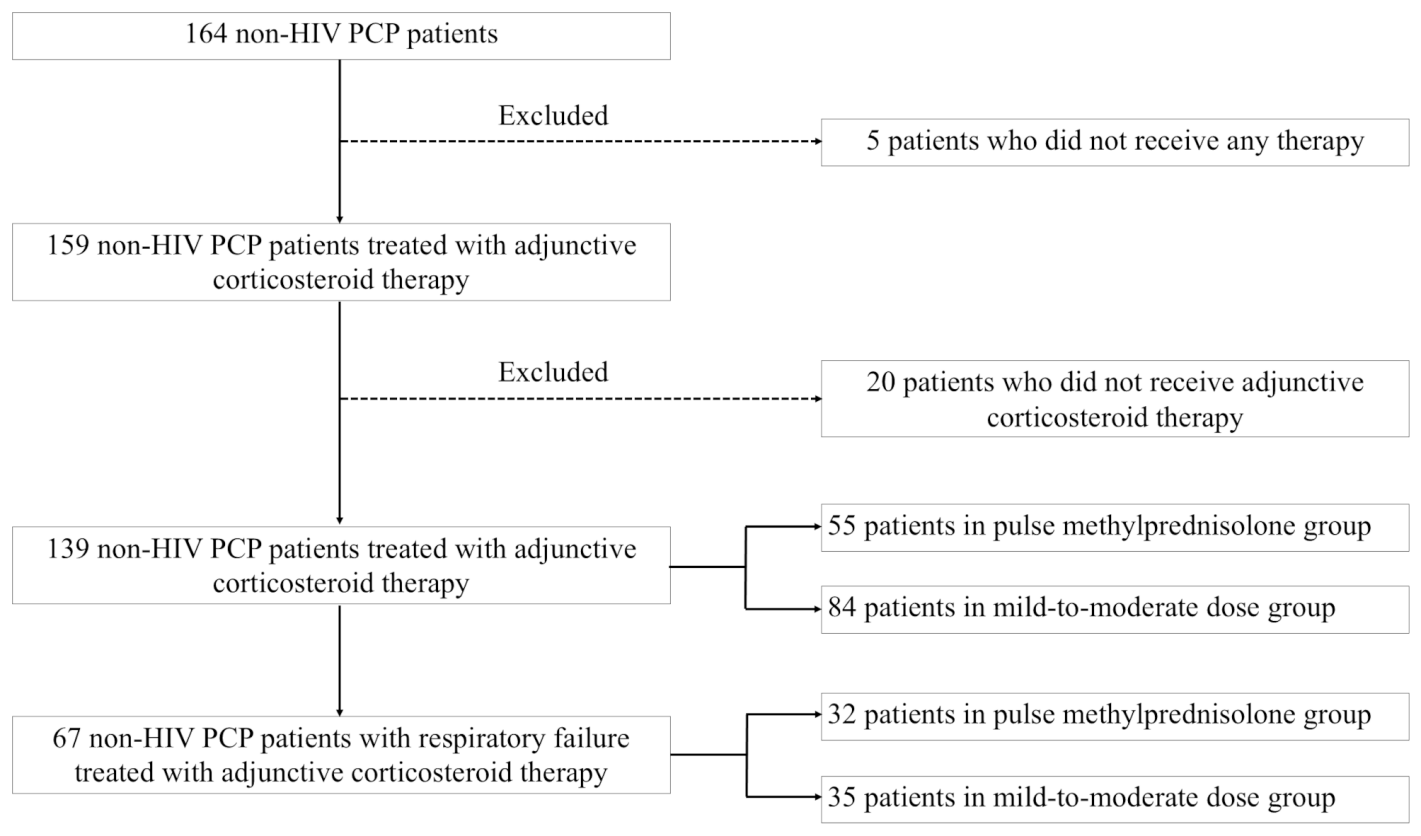
Flowchart showing study design and patient selection PCP, *Pneumocystis jirovecii* pneumonia; HIV, human immunodeficiency virus

A flowchart of the study is shown in Fig. 1. Data were collected from 164 patients diagnosed with non-HIV PCP. Five patients who did not receive any therapy and 20 patients who did not receive adjunctive corticosteroid therapy were excluded. Therefore, 139 patients with non-HIV PCP were included in this study and were categorised into two groups: 55 in the pulse methylprednisolone group and 84 in the mild-to-moderate dose group. In the subgroup analysis, 67 patients with respiratory failure were selected from the 139 patients with non-HIV PCP. The patients were classified into two groups: 32 in the pulse methylprednisolone group and 35 in the mild-to-moderate dose group. The descriptive data on the patient backgrounds and outcomes of the 20 cases that did not receive adjunctive corticosteroid therapy and were excluded from this study, and for all 139 cases that received adjunctive corticosteroid therapy and were included in the analysis, are provided in Supplementary Tables 1 and 2.

The unadjusted and adjusted baseline characteristics of the study participants are shown in Table 1. In the unadjusted cohort, sex, weight, hospital, presence of malignancies, solid tumours, connective tissue disease, or interstitial pneumonia, and use of corticosteroids, immunosuppressants, biologics, or anticancer agents were imbalanced between the two groups because the SMD was > 0.1. Among the laboratory results, haemoglobin, serum albumin, lactate dehydrogenase, creatinine levels, hypotension, and respiratory status were imbalanced across the two groups. In the adjusted cohort, balance was achieved for the adjustment variables, including age, sex, hospital, presence of malignancies, interstitial pneumonia or connective tissue disease, serum albumin and lactate dehydrogenase levels, serum creatinine clearance, and respiratory status, with an SMD of less than 0.1. Additionally, other variables, such as haemoglobin and serum sodium levels and hypotension, were also well balanced, with SMD values below 0.1.

**Table 1:** Baseline characteristics of participants

Variables	Unadjusted cohort			Adjusted cohort		
	Pulse methylprednisolone *n* = 55	Mild-to-moderate dose *n* = 84	SMD	Pulse methylprednisolone *n* = 25.8	Mild-to-moderate dose *n* = 25.8	SMD
Age (years)	70.67 (± 11.33)	70.21 (± 10.52)	0.042	70.54 (± 11.15)	70.54 (± 10.38)	< 0.001
Female	25 (45.5)	50 (59.5)	0.285	12.6 (48.8)	12.6 (48.8)	< 0.001
Weight (kg)	52.24 (± 10.80)	53.55 (± 11.99)	0.115	52.03 (± 11.12)	54.70 (± 12.46)	0.226
Hospital			0.280			< 0.001
Kameda Medical Center	35 (63.6)	49 (58.3)		16.6 (64.5)	16.6 (64.5)	
Seirei Hamamatsu General Hospital	13 (23.6)	29 (34.5)		6.8 (26.4)	6.8 (26.4)	
Seirei Mikatahara General Hospital	7 (12.7)	6 (7.1)		2.3 (9.0)	2.3 (9.0)	
Underlying disease						
Malignancies	15 (27.3)	13 (15.5)	0.291	5.6 (21.8)	5.6 (21.8)	< 0.001
Haematological malignancies	5 (9.1)	6 (7.1)	0.071	1.8 (7.1)	2.8 (10.9)	0.132
Solid tumours	11 (20.0)	8 (9.5)	0.299	4.0 (15.5)	3.0 (11.7)	0.111
Connective tissue disease	34 (61.8)	68 (81.0)	0.433	17.9 (69.2)	17.9 (69.2)	< 0.001
Interstitial pneumonia	23 (41.8)	13 (15.5)	0.609	7.3 (28.2)	7.3 (28.2)	< 0.001
Immunosuppressive agents used						
Corticosteroid	35 (63.6)	58 (69.0)	0.115	15.8 (61.2)	18.5 (71.5)	0.221
Immunosuppressant	32 (58.2)	55 (65.5)	0.151	16.5 (64.0)	12.8 (49.7)	0.293
Biologics	9 (16.4)	22 (26.2)	0.242	6.4 (24.7)	4.7 (18.2)	0.158
Anticancer agent	12 (21.8)	4 (4.8)	0.519	4.3 (16.8)	1.5 (5.8)	0.351
Blood biochemistry						
Haemoglobin (g/dL)	11.22 (± 2.13)	11.57 (± 1.93)	0.170	11.29 (± 2.06)	11.37 (± 1.99)	0.040
Platelet count (×10⁴/µL)	21.69 (± 9.48)	22.55 (± 10.95)	0.085	21.26 (± 9.21)	22.76 (± 11.72)	0.142
Albumin (g/dL)	2.75 (± 0.64)	3.06 (± 0.61)	0.500	2.88 (± 0.65)	2.88 (± 0.61)	< 0.001
Lactate dehydrogenase (IU/L)	440.15 (± 153.04)	386.81 (± 169.50)	0.330	415.52 (± 132.00)	415.52 (± 195.95)	< 0.001
Serum sodium (mEq/L)	137.73 (± 4.89)	137.85 (± 3.68)	0.027	137.49 (± 4.54)	137.65 (± 4.19)	0.037
Serum potassium (mEq/L)	4.17 (± 0.59)	4.22 (± 0.46)	0.100	4.14 (± 0.57)	4.21 (± 0.45)	0.142
Creatinine (mg/dL)	0.99 (± 0.85)	1.10 (± 1.31)	0.101	0.99 (± 0.78)	1.31 (± 1.64)	0.247
Creatinine clearance (mL/min)	60.79 (± 29.37)	61.39 (± 28.16)	0.021	60.40 (± 30.25)	60.40 (± 30.85)	< 0.001
Disturbed consciousness	1 (1.8)	1 (1.2)	0.052	0.2 (0.7)	0.7 (2.7)	0.160
Hypotension (systolic pressure < 90 mmHg)	2 (3.6)	1 (1.2)	0.160	0.4 (1.6)	0.5 (2.1)	0.033
Respiratory status			0.335			< 0.001
Without oxygen	23 (41.8)	49 (58.3)		11.9 (46.1)	11.9 (46.1)	
Administration of oxygen	31 (56.4)	34 (40.5)		13.6 (52.9)	13.6 (52.9)	
1–4 L/min	11 (20.0)	28 (33.3)		4.4 (17.0)	10.2 (39.4)	
5–10 L/min	12 (21.8)	1 (1.2)		5.4 (21.0)	0.7 (2.6)	
11–15 L/min	8 (14.5)	5 (6.0)		3.8 (14.9)	2.8 (10.9)	
Mechanical ventilation	1 (1.8)	1 (1.2)		0.3 (1.1)	0.3 (1.1)	
Initial treatment			0.200			0.277
Trimethoprim-Sulfamethoxazole	51 (92.7)	73 (86.9)		24.2 (94.0)	22.2 (86.1)	
Pentamidine	1 (1.8)	2 (2.4)		0.4 (1.6)	0.5 (2.0)	
Atovaquone	3 (5.5)	9 (10.7)		1.1 (4.5)	3.1 (11.9)	

Categorical variables are presented as *n* (%), and continuous variables are presented as mean ± standard deviation

Abbreviations: SMD = standardised mean difference

The clinical outcomes of the unadjusted and adjusted cohorts are summarised in Table 2. In the unadjusted cohort, the 30-day mortality rates were 21.8% for the pulse methylprednisolone group and 8.3% for the mild-to-moderate dose group. The 180-day mortality rates were 41.8% in the pulse methylprednisolone group and 14.3% in the mild-to-moderate dose group. In the adjusted cohort, there were no significant differences (30-day mortality: 14.2%, pulse methylprednisolone group and 15.5% in the mild-to-moderate dose group; and 180-day mortality: 33.5%, pulse methylprednisolone group and 27.3%, mild-to-moderate dose group).

**Table 2:** Summary of clinical outcomes

Variables	Unadjusted cohort			Adjusted cohort		
	Pulse methylprednisolone *n* = 55	Mild-to-moderate dose *n* = 84	*P*	Pulse methylprednisolone *n* = 25.8	Mild-to-moderate dose *n* = 25.8	*P*
Primary endpoint						
30-day mortality	12 (21.8)	7 (8.3)	0.044	3.7 (14.2)	4.0 (15.5)	0.850
Secondary endpoint						
180-day mortality	23 (41.8)	12 (14.3)	0.001	8.6 (33.5)	7.1 (27.3)	0.516

Categorical variables are presented as *n* (%)

Subsequently, we performed a subgroup analysis of patients with respiratory failure. The unadjusted and adjusted baseline characteristics of the subgroups are presented in Table 3. In the unadjusted cohort, sex, weight, hospital, presence of malignancies, haematological malignancies, solid tumours, connective tissue disease, or interstitial pneumonia, and the use of immunosuppressants, biologics, or anticancer agents were imbalanced. Among the laboratory results, haemoglobin, serum albumin, lactate dehydrogenase, serum sodium, creatinine, and creatinine clearance levels were imbalanced, as were disturbed consciousness and initial treatment. In the adjusted cohort, the adjustment variables—including age, sex, hospital, presence of malignancies, interstitial pneumonia or connective tissue disease, serum albumin, lactate dehydrogenase, serum creatinine clearance, and respiratory status—were well balanced, with SMD values below 0.1. Furthermore, other variables, such as the use of biologics and haemoglobin, serum sodium, and serum potassium levels, also showed good balance, with SMD values under 0.1.

**Table 3:** Baseline characteristics of patients in the subgroup analysis limited to respiratory failure

Variables	Unadjusted cohort			Adjusted cohort		
	Pulse methylprednisolone *n* = 32	Mild-to-moderate dose *n* = 35	SMD	Pulse methylprednisolone *n* = 13.3	Mild-to-moderate dose *n* = 13.3	SMD
Age (years)	71.56 (± 11.84)	71.46 (± 9.49)	0.010	71.79 (± 12.77)	71.79 (± 9.86)	< 0.001
Female	14 (43.8)	19 (54.3)	0.212	6.1 (45.5)	6.1 (45.5)	< 0.001
Weight (kg)	52.56 (± 9.76)	54.43 (± 12.16)	0.169	52.61 (± 10.51)	55.76 (± 12.36)	0.275
Hospital			0.249			< 0.001
Kameda Medical Center	27 (84.4)	28 (80.0)		11.2 (84.1)	11.2 (84.1)	
Seirei Hamamatsu General Hospital	5 (15.6)	6 (17.1)		2.1 (15.9)	2.1 (15.9)	
Seirei Mikatahara General Hospital	0 (0.0)	1 (2.9)		0.0 (0.0)	0.0 (0.0)	
Underlying disease						
Malignancies	12 (37.5)	6 (17.1)	0.469	3.4 (25.6)	3.4 (25.6)	< 0.001
Haematological malignancies	4 (12.5)	3 (8.6)	0.128	0.9 (6.7)	2.0 (14.7)	0.263
Solid tumours	9 (28.1)	3 (8.6)	0.522	2.7 (19.9)	1.5 (10.9)	0.251
Connective tissue disease	16 (50.0)	26 (74.3)	0.517	8.2 (61.8)	8.2 (61.8)	< 0.001
Interstitial pneumonia	10 (31.2)	7 (20.0)	0.260	3.0 (22.8)	3.0 (22.8)	< 0.001
Immunosuppressive agents used						
Corticosteroid	24 (75.0)	26 (74.3)	0.016	11.0 (82.9)	8.9 (67.1)	0.371
Immunosuppressant	13 (40.6)	18 (51.4)	0.218	6.2 (46.4)	5.3 (39.6)	0.136
Biologics	2 (6.2)	6 (17.1)	0.344	1.4 (10.5)	1.6 (11.7)	0.041
Anticancer agent	10 (31.2)	2 (5.7)	0.697	2.9 (21.9)	1.0 (7.4)	0.420
Blood biochemistry						
Haemoglobin (g/dL)	11.21 (± 2.28)	11.57 (± 2.04)	0.167	11.47 (± 2.26)	11.33 (± 2.02)	0.068
Platelet count (×10⁴/µL)	22.54 (± 10.01)	21.63 (± 11.51)	0.085	23.11 (± 10.32)	20.89 (± 11.58)	0.203
Albumin (g/dL)	2.52 (± 0.49)	2.81 (± 0.61)	0.529	2.67 (± 0.48)	2.67 (± 0.58)	< 0.001
Lactate dehydrogenase (IU/L)	458.38 (± 167.76)	377.00 (± 169.81)	0.482	428.69 (± 143.55)	428.69 (± 213.10)	< 0.001
Serum sodium (mEq/L)	138.62 (± 5.53)	137.83 (± 4.15)	0.163	138.44 (± 4.52)	138.08 (± 4.22)	0.083
Serum potassium (mEq/L)	4.11 (± 0.65)	4.11 (± 0.40)	0.015	4.11 (± 0.60)	4.12 (± 0.41)	0.016
Creatinine (mg/dL)	0.89 (± 0.50)	1.35 (± 1.88)	0.333	0.96 (± 0.62)	1.53 (± 2.17)	0.353
Creatinine clearance (mL/min)	62.68 (± 28.42)	59.38 (± 30.07)	0.113	60.72 (± 29.75)	60.72 (± 32.05)	< 0.001
Disturbed consciousness	0 (0.0)	1 (2.9)	0.243	0.0 (0.0)	0.6 (4.3)	0.301
Hypotension (systolic pressure < 90 mmHg)	1 (3.1)	1 (2.9)	0.016	0.2 (1.8)	0.5 (3.7)	0.117
Respiratory status			0.016			< 0.001
Administration of oxygen	31 (96.9)	34 (97.1)		13.0 (97.9)	13.0 (97.9)	
1–4 L/min	11 (34.4)	28 (80.0)		4.1 (30.6)	9.4 (70.5)	
5–10 L/min	12 (37.5)	1 (2.9)		5.0 (37.6)	0.6 (4.6)	
11–15 L/min	8 (25.0)	5 (14.3)		4.0 (29.8)	3.0 (22.8)	
Mechanical ventilation	1 (3.1)	1 (2.9)		0.3 (2.1)	0.3 (2.1)	
Initial treatment			0.508			0.544
Trimethoprim-Sulfamethoxazole	32 (100.0)	31 (88.6)		13.3 (100.0)	11.6 (87.1)	
Pentamidine	0 (0.0)	0 (0.0)		0 (0.0)	0 (0.0)	
Atovaquone	0 (0.0)	4 (11.4)		0 (0.0)	1.7 (12.9)	

Categorical variables are presented as *n* (%), and continuous variables are presented as mean ± standard deviation

Abbreviations: SMD = standardised mean difference

The clinical outcomes of both cohorts are shown in Table 4. In the unadjusted cohort, the 30-day mortality rates were 25.0% for the pulse methylprednisolone group and 17.1% for the mild-to-moderate dose group. The 180-day mortality rates were 50.0% in the pulse methylprednisolone group and 25.7% in the mild-to-moderate dose group. No significant differences were observed between the two groups in the adjusted cohort (30-day mortality: 20.7%, pulse methylprednisolone group and 27.7%, mild-to-moderate dose group; and 180-day mortality: 44.0%, pulse methylprednisolone group and 36.3%, mild-to-moderate dose group).

**Table 4:** Clinical outcomes of the subgroup analysis limited to respiratory failure

Variables	Unadjusted cohort			Adjusted cohort		
	Pulse methylprednisolone *n* = 32	Mild-to-moderate dose *n* = 35	*P*	Pulse methylprednisolone *n* = 13.3	Mild-to-moderate dose *n* = 13.3	*P*
Primary endpoint						
30-day mortality	8 (25.0)	6 (17.1)	0.625	2.8 (20.7)	3.7 (27.7)	0.565
Secondary endpoint						
180-day mortality	16 (50.0)	9 (25.7)	0.072	5.9 (44.0)	4.8 (36.3)	0.583

Categorical variables are presented as *n* (%)

The results of the sensitivity analysis using log-rank test and Cox proportional hazards analysis are shown in Supplementary Tables 3 and 4. In the overall analysis, the adjusted HR for pulse methylprednisolone, with mild-to-moderate dose therapy as the control (HR = 1), was 0.86 (95% confidence interval 0.48–1.51, *P* = 0.591), which was not statistically significant (Supplementary Table 3). Similarly, in the analysis limited to cases with respiratory failure, the HR for pulse methylprednisolone was 1.19 (95% confidence interval 0.56–2.51, *P* = 0.653), which was also not statistically significant (Supplementary Table 4). The Kaplan-Meier curves are shown in Supplementary Fig. 1 (overall analysis) and 2 (analysis limited to cases with respiratory failure).

## Discussion

In this multicentre, retrospective, observational cohort study of patients with non-HIV PCP treated with adjunctive corticosteroid therapy, we evaluated the efficacy of pulse methylprednisolone compared to mild-to-moderate steroid doses. No significant differences were observed between the two groups in terms of the 30- or 180-day mortality rates after adjusting for patient background using the overlap weighting method with propensity scores. Additionally, similar results were obtained in the sensitivity analysis using the Cox proportional hazards model. To our knowledge, this study is the first to compare pulse methylprednisolone with other doses for the treatment of non-HIV PCP using hospital-based detailed clinical information after adjusting for patient background. In contrast, some reports exist on the appropriateness of dosage and the effectiveness of adjunctive corticosteroid therapy in HIV PCP.

Several randomised controlled trials and a meta-analysis have provided evidence that adjunctive corticosteroid therapy reduces the need for mechanical ventilation and mortality in patients with HIV-associated PCP and hypoxemia [8–11]. A Cochrane meta-analysis, which evaluated six randomised controlled trials on the efficacy of adjunctive steroids for moderate to severe HIV-associated PCP, found that compared to placebo, patients receiving adjunctive steroids had a lower risk of overall mortality at 1 month (risk ratio 0.56, 95% CI 0.32–0.98) and at 3–4 months (risk ratio 0.59, 95% CI 0.41–0.85) of follow-up [11]. Therefore, international guidelines recommend an initial dose of 80 mg/day of adjunctive corticosteroids for adult patients with moderate-to-severe HIV-associated PCP, defined as arterial oxygen partial pressure < 70 mmHg or alveolar-arterial gradient > 35 mmHg on room air [7, 11, 30].

In contrast to HIV-associated PCP, there are limited data on the effectiveness of adjunctive corticosteroid therapy in patients with non-HIV-associated PCP. The American Thoracic Society’s Statement on the treatment of fungal infections in adult pulmonary patients recommends a corticosteroid treatment regimen for patients with moderate to severe non-HIV-associated PCP, similar to that used for HIV-associated PCP, as previously described [5]. However, this recommendation for adjunctive corticosteroid therapy in non-HIV PCP is primarily based on studies conducted in HIV-associated PCP [10, 30], and there is a lack of solid evidence from randomised controlled trials specifically focusing on PCP in patients without HIV. The current recommendation lacks a robust foundation of evidence in the non-HIV population [22]. As discussed in the introduction, there is an increasing body of evidence supporting the effectiveness of adjunctive glucocorticoid treatment in patients with respiratory failure. However, the optimal dosage of glucocorticoids for PCP treatment in patients without HIV remains inadequately explored [13, 14]. In this multicentre study, we found no significant difference in the 30- and 180-day mortality rates between pulse methylprednisolone and mild-to-moderate steroid doses after adjusting for patient background. Additionally, no significant association was observed in the subgroup analysis of patients with respiratory failure. These findings suggest that pulse methylprednisolone may not be necessary as an adjunctive steroid in non-HIV PCP, and mild-to-moderate steroid doses could be sufficient. Nevertheless, it is important to note that further large prospective cohort studies and randomised controlled trials are needed to confirm these results.

The strength of this study lies in its multicentre design, which has a large number of patients adjusted for background. In clinical practice, higher doses of corticosteroids tend to be administered in more severe cases, and there may be a selection bias in patients treated with adjunctive corticosteroids. In this study, the respiratory status tended to be worse in the pulse methylprednisolone group between the two groups before adjustment for patient background. Patients with interstitial lung disease may also be more likely to be selected for pulse methylprednisolone therapy due to suspected acute exacerbations [26], potentially leading to patient background bias. Indeed, there were more patients with interstitial pneumonia in the pulse methylprednisolone group before adjusting for patient characteristics. In this study, patient characteristics, including interstitial pneumonia, respiratory status, other prognostic factors (age, sex, serum albumin levels, serum lactate dehydrogenase levels, and the presence of malignancy), and connective tissue disease were adjusted using the overlap weighting method in propensity score analysis [15].

Our study had some limitations. First, this was a retrospective, observational study. Although we adjusted for confounding factors related to pulse methylprednisolone therapy and outcomes, such as interstitial pneumonia and prognostic factors, using propensity score analysis, we could not completely adjust for unknown confounding factors. Future prospective randomised trials are required to eliminate the influence of these confounders. Second, this study did not examine adverse events associated with corticosteroid use. Although the adverse events associated with systemic corticosteroid use (including hyperglycaemia, increased risk of coinfections, delirium, and ICU-acquired weakness) are generally recognised, data on the safety of adjunctive corticosteroid therapy for non-HIV PCP are lacking. The selection of the corticosteroid therapy dose should be based not only on the outcome but also on safety. Future studies are required to evaluate the safety of steroid use. Third, arterial blood gas analysis was not performed in many cases in the original dataset; thus, data on PaO_2_ and alveolar-arterial oxygen gradient (A-a O_2_ gradient) were missing, making it unfeasible to apply the standard severity classification for PCP [31]. Respiratory impairment is typically classified as mild (PaO_2_ ≥ 70 mmHg or A-a O_2_ gradient < 35 mmHg, or both), moderate (60 mmHg ≤ PaO_2_ < 70 mmHg or 35 mmHg ≤ A-a O_2_ gradient < 45 mmHg, or both), and severe (PaO_2_ < 60 mmHg or A-a O_2_ gradient ≥ 45 mmHg, or both). As substitute indicators of respiratory status, we used the implementation of oxygen supplementation, amount of oxygen administered, and need for mechanical ventilation. In Japan, oxygen is typically administered when the SpO_2_ approximately falls below 90–93%. Since an SpO_2_ of 90% corresponds to a PaO_2_ of around 60 mm Hg [32], we considered cases requiring oxygen supplementation as corresponding to the moderate-to-severe category. We conducted a subgroup analysis focusing on cases with respiratory failure (requiring oxygen or mechanical ventilation) and found no significant difference in mortality between the pulse methylprednisolone group and the mild to moderate dose steroid group, suggesting that the efficacy remains similar even when restricted to moderate-to-severe cases. Finally, this study did not directly compare outcomes between patients who received adjunctive corticosteroids and those who did not. Although recent observational studies suggest potential benefits of corticosteroids in non-HIV PCP [13, 14], the definitive impact of corticosteroids on patient outcomes remains a topic of ongoing debate. Descriptive data for patients who did not receive adjunctive corticosteroid therapy (the non-corticosteroid group) are provided in Supplementary Tables 1 and 2. Compared to patients who received adjunctive corticosteroid therapy (the corticosteroid group), fewer non-corticosteroid patients required oxygen therapy (25% vs. 46.8%); among those non-corticosteroid patients who did require oxygen, only low flow rates (1–4 L/min) were necessary. Additionally, key prognostic factors indicated milder disease in the non-corticosteroid group, with higher albumin levels (3.1 g/dL vs. 2.94 g/dL) and lower LDH levels (350 IU/L vs. 407 IU/L). Given these substantial differences in patient characteristics and the small sample size of the non-corticosteroid group, it was challenging to compare outcomes between the two groups. Consequently, this study focused on corticosteroid dosage rather than the presence or absence of steroid use. Nevertheless, with the increasing interest in low-dose corticosteroids for improving outcomes in respiratory infections, such as COVID-19 and severe community-acquired pneumonia [33], we believe our analysis comparing pulse methylprednisolone therapy with mild-to-moderate dose therapy offers valuable clinical insights.

## Conclusions

Pulse methylprednisolone therapy was not observed to be more effective than a mild-to-moderate dose regimen in the treatment of patients with non-HIV PCP after adjusting for patient characteristics. A mild-to-moderate dose of adjunctive corticosteroid may be sufficient for treating patients with non-HIV PCP. Further studies, including large-scale prospective cohort studies and randomised controlled studies, are required to confirm our results.

## Electronic supplementary material

Below is the link to the electronic supplementary material.

Supplementary Material 1

Supplementary Material 2

## Data Availability

Data pertaining to this study will be available by the corresponding author upon reasonable request.

## Declarations

### Reporting Checklist

The authors have completed the STROBE reporting checklist (https://www.strobe-statement.org/) (submitted as a supplementary file).

## Abbreviations

A-a O_2_ gradient: Alveolar-arterial oxygen gradient
HIV: Human immunodeficiency virus
HR: Hazard risk
ICU: Intensive care unit
PCP: *Pneumocystis jirovecii* pneumonia
SMD: Standardised mean difference
