# Supplementary material for: Effectiveness of pulse methylprednisolone in patients with non-human immunodeficiency virus pneumocystis pneumonia: a multicentre, retrospective registry-based cohort study
Source: BMC Infect Dis. 2024 Nov 2;24:1233. doi: 10.1186/s12879-024-10151-3 (PMC11531689; doi:10.1186/s12879-024-10151-3)

Supplementary Data

Supplementary Table 1. Patient characteristics in with and without adjunctive corticosteroid therapy groups

| Variables | Without adjunctive corticosteroid therapy  n = 20 | With adjunctive corticosteroid therapy  n = 139 |
| --- | --- | --- |
| Age (years) | 72.05 (±10.39) | 70.40 (±10.81) |
| Female | 11 (55.0) | 75 (54.0) |
| Weight (kg) | 49.20 (±11.01) | 53.03 (±11.51) |
| Hospital |  |  |
| Kameda Medical Center | 8 (40.0) | 84 (60.4) |
| Seirei Hamamatsu General Hospital | 9 (45.0) | 42 (30.2) |
| Seirei Mikatahara General Hospital | 3 (15.0) | 13 (9.4) |
| Underlying disease |  |  |
| Malignancies | 5 (25.0) | 28 (20.1) |
| Haematological malignancies | 4 (20.0) | 11 (7.9) |
| Solid tumours | 1 (5.0) | 19 (13.7) |
| Connective tissue disease | 14 (70.0) | 102 (73.4) |
| Interstitial pneumonia | 5 (25.0) | 36 (25.9) |
| Immunosuppressive agents used |  |  |
| Corticosteroid | 9 (45.0) | 93 (66.9) |
| Immunosuppressant | 13 (65.0) | 87 (62.6) |
| Biologics | 9 (45.0) | 31 (22.3) |
| Anticancer agent | 2 (10.0) | 16 (11.5) |
| Blood biochemistry |  |  |
| Haemoglobin (g/dL) | 11.25 (±2.22) | 11.43 (±2.01) |
| Platelet count (×10⁴/μL) | 17.75 (±8.60) | 22.21 (±10.37) |
| Albumin (g/dL) | 3.10 (±0.58) | 2.94 (±0.64) |
| Lactate dehydrogenase (IU/L) | 350.65 (±97.49) | 407.91 (±164.71) |
| Serum sodium (mEq/L) | 136.15 (±4.33) | 137.80 (±4.18) |
| Serum potassium (mEq/L) | 4.19 (±0.51) | 4.20 (±0.51) |
| Creatinine (mg/dL) | 1.30 (±2.66) | 1.06 (±1.15) |
| Creatinine clearance (mL/min) | 58.83 (±22.87) | 61.15 (±28.54) |
| Disturbed consciousness | 0 (0.0) | 2 (1.4) |
| Hypotension (systolic pressure <90 mmHg) | 0 (0.0) | 3 (2.2) |
| Respiratory status |  |  |
| Without oxygen | 15 (75.0) | 72 (51.8) |
| Administration of oxygen | 5 (25.0) | 65 (46.8) |
| 1−4 L/min | 5 (25.0) | 39 (28.1) |
| 5−10 L/min | 0 (0.0) | 13 (9.4) |
| 11−15 L/min | 0 (0.0) | 13 (9.4) |
| Mechanical ventilation | 0 (0.0) | 2 (1.4) |
| Initial treatment |  |  |
| Trimethoprim-Sulfamethoxazole | 19 (95.0) | 124 (89.2) |
| Pentamidine | 1 (5.0) | 3 (2.2) |
| Atovaquone | 0 (0.0) | 12 (8.6) |

Supplementary Table 2. Outcomes of patients with and without adjunctive corticosteroid therapy

| Variables | Without adjunctive corticosteroid therapy  n = 20 | With adjunctive corticosteroid therapy  n = 139 |
| --- | --- | --- |
| Primary endpoint |  |  |
| 30-day mortality | 1 (5.0) | 19 (13.7) |
| Secondary endpoint |  |  |
| 180-day mortality | 2 (10.0) | 35 (25.2) |

Supplementary Table 3. The effect of pulse methylprednisolone on mortality risk based on log-rank test and Cox proportional hazards model (n=139)

|  | Unadjusted cohort | | Adjusted cohort* | |
| --- | --- | --- | --- | --- |
|  | HR (95%CI) | P value | HR (95%CI) | P value |
| Mild-to-moderate dose | 1.00 (reference) |  | 1.00 (reference) |  |
| Pulse methylprednisolone | 2.08 (1.23–3.52) | 0.006 | 0.86 (0.48–1.51) | 0.591 |

HR, hazard risk: CI, confidence interval

* Adjusted using overlap weighting based on the propensity score, accounting for age, sex, hospital, serum albumin levels, lactate dehydrogenase levels, respiratory status, creatinine clearance, and the presence of malignancy, interstitial pneumonia, and connective tissue disease.

Supplementary Table 4. The effect of pulse methylprednisolone on mortality risk based on log-rank test and Cox proportional hazards model in the subgroup analysis limited to respiratory failure (n=67)

|  | Unadjusted cohort | | Adjusted cohort* | |
| --- | --- | --- | --- | --- |
|  | HR (95%CI) | P value | HR (95%CI) | P value |
| Mild-to-moderate dose | 1.00 (reference) |  | 1.00 (reference) |  |
| Pulse methylprednisolone | 2.28 (1.12–4.61) | 0.023 | 1.19 (0.56–2.51) | 0.653 |

HR, hazard risk: CI, confidence interval

* Adjusted using overlap weighting based on the propensity score, accounting for age, sex, hospital, serum albumin levels, lactate dehydrogenase levels, respiratory status, creatinine clearance, and the presence of malignancy, interstitial pneumonia, and connective tissue disease.

Supplementary Figure 1. Kaplan-Meier curve for the effect of pulse methylprednisolone on mortality risk (n=139)


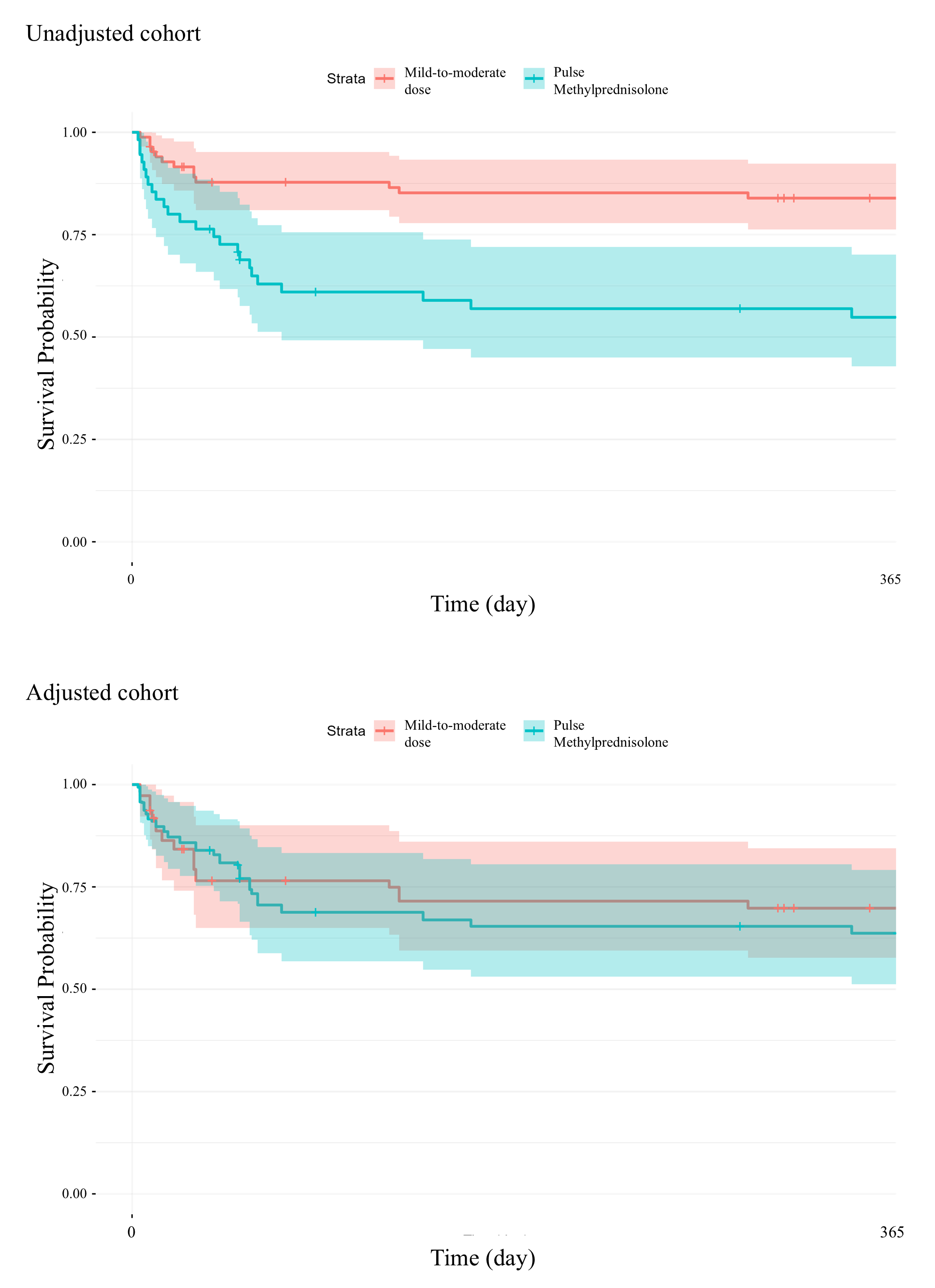


Supplementary Figure 2. Kaplan-Meier curve for the effect of pulse methylprednisolone on mortality risk in patients with respiratory failure (n=67)


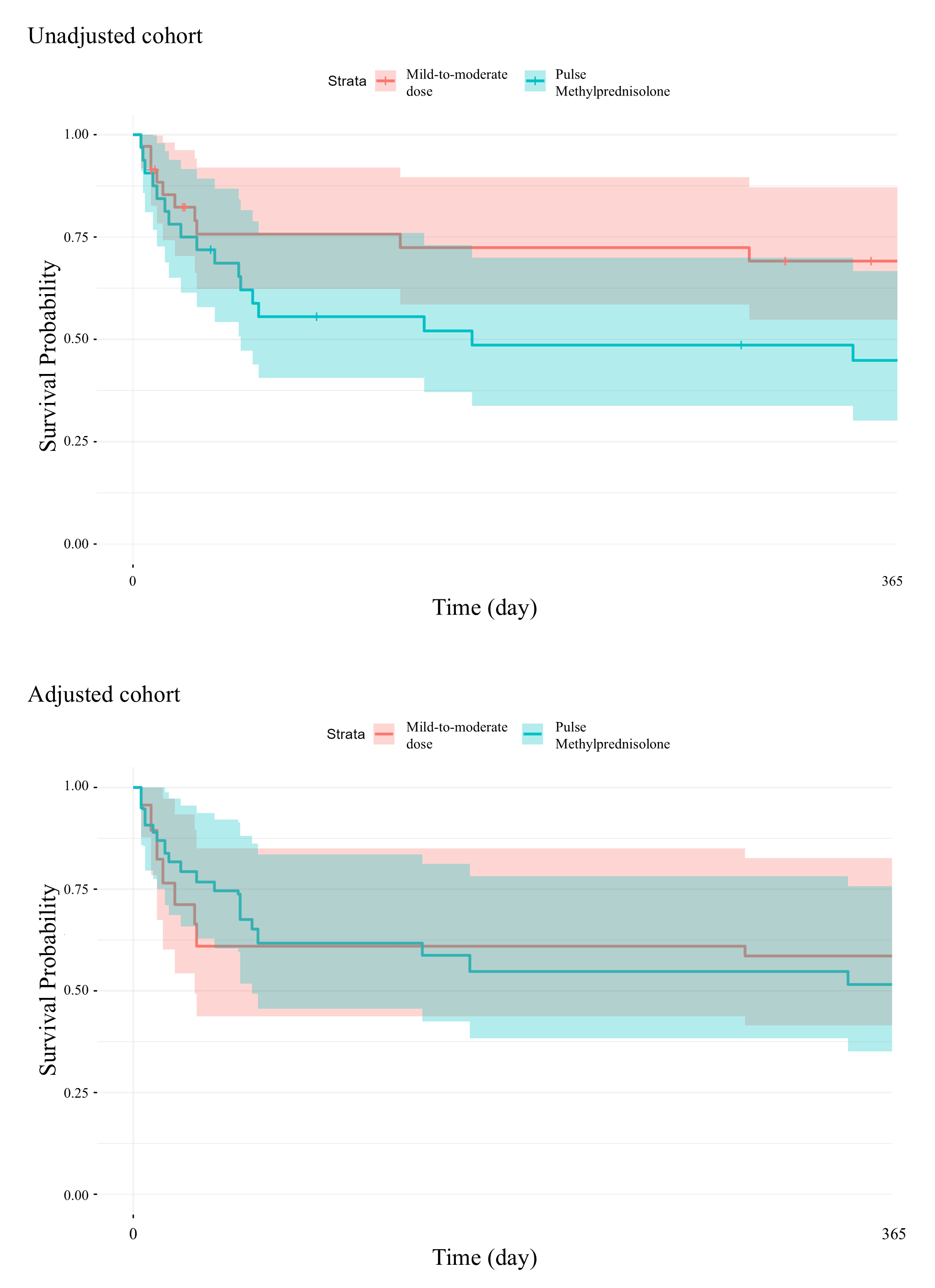

Supplement: Supplementary file 1 — Supplementary Material 1 [file 12879_2024_10151_MOESM1_ESM.docx]
